# Six-minute walk test protocol variations in low-resource settings – A scoping review

**DOI:** 10.4102/sajp.v77i1.1549

**Published:** 2021-06-24

**Authors:** Brittany L. Fell, Susan Hanekom, Martin Heine

**Affiliations:** 1 Division of Physiotherapy, Faculty of Medicine and Health Sciences, Stellenbosch University, Cape Town, South Africa; 2 Institute of Sport and Exercise Medicine, Faculty of Medicine and Health Sciences, Stellenbosch University, Cape Town, South Africa

**Keywords:** rehabilitation, walking test, functional capacity, outcome assessment, non-communicable diseases

## Abstract

**Background:**

The 6-min walk test (6MWT) is a validated tool, of submaximal intensity, used to objectively measure functional exercise capacity. In 2002, the American Thoracic Society (ATS) developed guidelines on standardising the implementation of the 6MWT. Despite the relative ease of conducting the 6MWT as per these guidelines, adaptations are implemented.

**Objectives:**

Identify (1) what 6MWT adaptations to the ATS guidelines have been described in low-resource settings (LRS), (2) the purpose of the adapted 6MWT and (3) the reported argumentation for making these adaptations in relation to the specific context.

**Methods:**

Five databases were searched from inception until February 2021. Studies that adapted and conducted the 6MWT in LRS were included. Data concerning the study source, participants, 6MWT: purpose, variations, outcome and rationale were extracted.

**Results:**

A total of 24 studies were included. The majority of studies (*n* = 18; 75%) were conducted in lower-middle income countries. The most common adaptation implemented was variation to course length. Eight studies provided a rationale for adapting the 6MWT. Space constraint was the most common reason for adaptation.

**Conclusion:**

The most common reason (space constraints) for adapting the 6MWT in LRS was addressed through adaptations in course length and/or configuration. The results of this review suggest that the value of the ATS-guided 6MWT in LRS may need to be re-evaluated.

**Clinical implications:**

Using adapted forms of the 6MWT may lead to an underestimation of a patient’s abilities, misinformed discharge and developing inappropriate exercise programmes. Additionally, diverting from ATS guidelines may affect the continuity of care.

## Introduction

The 6-min walk test (6MWT) is a validated tool, of submaximal intensity, used to objectively measure functional exercise capacity across numerous pathologies including patients with multiple co-morbidities (Chetta et al. 2006; Dourado et al. 2009). This field test is conducted by having a participant walk a fixed lap length over a set time period of 6 min (Chetta et al. 2006). In 2002, the American Thoracic Society (ATS) developed and published guidelines on how to implement the test in a standardised manner (Crapo, Enright & Zeballos 2002). These guidelines aim to promote consistent clinical application of the test, thereby allowing comparisons, globally, across studies (Ubuane et al. 2018).

The ATS guidelines stipulate that the test is conducted indoors, on a hard-flat surface, using a 30 m (100 ft.) straight path (i.e. walkway or lap length), marked with cones on each end. Participants are subsequently instructed to walk as far as they can in 6 min, and standard phrases of encouragement are used at every minute mark. The 6-min walk distance (6MWD), the primary outcome derived from the 6MWT, is calculated by adding the number of completed laps with the distance of the unfinished lap the participant was able to achieve by the end of the 6 min (Bellet et al. 2011). The 6MWD can be interpreted by comparing it to a predicted normative value, using previously established reference equations or by using it as an absolute value for comparison to a previously completed test by the same participant (Ben Saad et al. 2009). Subsequently, there are many ways one can use the 6MWD in clinical practice or research.

Individualised exercise is the evidence-based cornerstone of any comprehensive rehabilitation programme for those with non-communicable disease, in particular those related to lifestyle (e.g. diabetes, cardiovascular disease). Improvements in physical function, through exercise, have been linked to reductions in premature mortality and morbidity (Van der Leeden et al. 2018) and clinical risk markers as well as improvements in quality of life (QOL), (Anderson & Durstine 2019) amongst others. For instance, patients with chronic obstructive pulmonary disease (COPD) have shown improvement in QOL and a reduction in their symptoms when following a low-to-moderate intensity exercise programme. However, greater physiological responses like increased exercise capacity and decreased ventilatory demand are noted when exercise programmes are of higher intensity (Rodrigues et al. 2016). This emphasises the need for patient assessment to help guide clinicians in developing patient-specific, yet appropriate, exercise programmes.

Whilst the gold standard for the objective measurement of maximal functional capacity is cardiopulmonary exercise testing (CPET), (Ubuane et al. 2018), one can argue that this resource-heavy (i.e. equipment, training) test is often not feasible or necessary. Conversely, the 6MWT is often recommended as the field test of choice in day-to-day clinical practice. Reasons for this include its applicability in various populations, physiological systems and settings. Additionally, the test is easy to perform, not time-consuming, does not require additional equipment or special skills and is also more representative of the exertion of everyday activities (Bellet et al. 2011; Chetta et al. 2006; Ubuane et al. 2018).

However, despite the relative ease of conducting the 6MWT in a standardised way, particularly in comparison to CPET, adaptations to the ‘ATS-guided 6MWT’ are being implemented (Sciurba et al. 2003). Typically, these adaptations may include changes to walkway distance and configuration, the inclusion of a practice run or test instructions, and encouragement, etc. Subsequently, the 6MWD may be influenced by these adaptations, (Barnett et al. 2016; Casillas et al. 2013), for instance, changes in gait speed, pacing strategy adopted and number of turns made to complete the test (Barnett et al. 2016; Ng et al. 2013). For example, Beekman et al. (2013) showed in a study involving patients with COPD that reducing pathway distance from 30 m to 10 m resulted in an average 49.5 m shorter distance. The difference in 6MWD between the two pathway distances was attributed to the increased number of turns on the 10 m pathway, and subsequently less time spent at optimal walking pace (Beekman et al. 2013). This discrepancy in 6MWD, in this case because of a difference in pathway length, may have clinical implications. Firstly, if one would compare the 10 m results to establish reference equations developed using ATS guidelines, it is likely that the patient’s functional abilities will be markedly underestimated. Secondly, the shorter distance achieved by the participants on the 10 m pathway (49.5 m) is larger than the reported minimum clinically important difference (MCID) of 35 m as reported by Puhan and colleagues, as well as the 30 m MCID reported by Polkey and colleagues (Polkey et al. 2013; Puhan et al. 2008). Thirdly, using data based on different 6MWT protocols interchangeably could lead to either premature or delayed discharge of patients as well as the prescription of an inappropriate exercise programme, amongst others.

In cases of a misinformed discharge (an extended hospital admission or early discharge), this may have a compounding effect, specifically in already resource-constrained or overburdened healthcare environments. Collectively, these examples highlight the importance of using standardised testing protocols, particularly in settings of low resources, (Van Zyl et al. 2021) where the gold standard for CPET is often unavailable and physical resources (e.g. space) may be limited.

The objective of our scoping review is therefore to identify: (1) what 6MWT adaptations to the ATS guidelines have been described in low-resource settings (LRS), (2) the purpose of the adapted 6MWT and (3) the reported argumentation for making these adaptations in relation to the specific context.

## Methods

Our scoping review was conducted in accordance with the framework provided by Arksey and O’Malley (Arksey & O’Malley 2005) and reported according to the PRISMA guidelines including scoping review extensions (Tricco et al. 2018) (see online Supplement 1).

### Data sources and search strategy

Five bibliographical databases were accessed through the Stellenbosch University Library: Cochrane Library, EBSCOhost (AfricaWide, Cumulative Index to Nursing and Allied Health Literature [CINAHL], Medline), PubMed, Scopus and Web of Science. An initial search was conducted (BF) from inception to 31 October 2019. Subsequently, the search was updated to include records up to 14 February 2021. Search terms included variations of the following main search terms: 6MWT, low-resource setting and developing countries (see online Supplement 2).

### Study selection

After the completion of the searches, removal of duplicates, titles and abstracts were independently screened by two reviewers (BF and MH). Any disagreements were discussed by the reviewers and a third reviewer was consulted (SH) in the case of irreconcilable disagreements. An identical procedure was followed for full text screening to determine final full text inclusions. All original research study designs that were available in English were considered. Included studies must have reported on and used an adapted or modified version of the 6MWT and conducted the test within the context of low-resource setting (Van Zyl et al. 2021). In the absence of a clear definition for LRS, within the context of an upper middle income country (UMIC) or high income country (HIC), studies were included based on the language (e.g. rural) used by the study in conjunction with online resources (detail on the location or context of a specific clinic) and purposeful discussion amongst the authors. All ages were considered for our review. After becoming familiar with the sources, decisions were made to exclude case studies and case series and any studies that performed the 6MWT according to ATS guidelines or did not report on the methods used. Despite searching online resources from inception, articles published before 2002 (year of published ATS guidelines) were excluded.

### Data extraction and synthesis

A data extraction form was developed, and revised as necessary, to extract relevant data from the included full-text articles and captured under the following headings: source, participants, reported 6MWT purpose, adaptations, outcomes and rationale. In case the rationale for making adaptations was unclear, corresponding authors were contacted. Data were extracted by the primary reviewer BF and verified independently by a second reviewer (MH). In the event of group data (e.g. male and female, or intervention and control group) being reported separately, an aggregated 6MWD mean and standard deviations were calculated for each study, using the formula provided in the Cochrane handbook; excluded data were reported separately for diseased groups with healthy controls (Higgins et al. 2019). In case of longitudinal studies, only baseline data were extracted.

### Concepts and context

#### Low-resource setting

In line with previous research, a low-resource setting was defined as a low-income country (LIC) or lower-middle income country (LMIC) as per the World Bank Criteria, (World Bank 2018), or in the event of an UMIC or HIC, an explicit statement indicating a LRS (e.g. rural, minority populations, poverty) was required (Heine et al. 2019).

#### Six-minute walk test adaptations

The test was considered adapted when it deviated from the 2002 ATS guidelines for the 6MWT, (Enright 2003) and grouped in the following categories:

- distance; shorter or longer than 30 m (100 ft.)
- configuration, that is, not a straight pathway
- venue; not indoors
- instructions or encouragement were not as provided by the ATS guidelines
- ‘other’ (e.g. treadmill).

### Ethical considerations

This article followed all ethical standards for research without direct contact with human or animal subjects.

## Results

**FIGURE 1:**
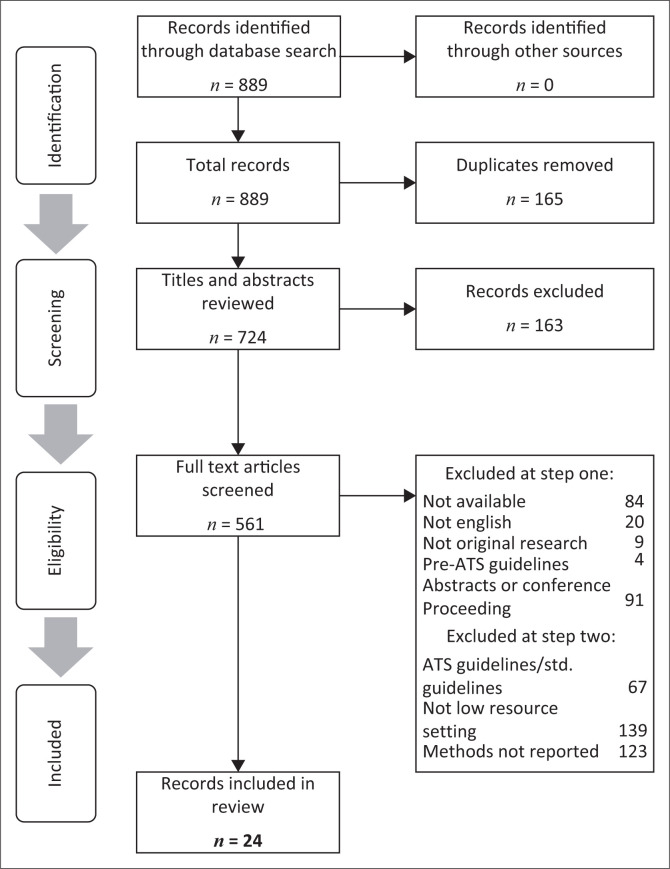
Preferred Reporting Items for Systematic Reviews and Meta-Analyses (PRISMA) flow chart of data synthesis.

The PRISMA flow chart is shown in Figure 1. Of the 564 records identified across the five databases, 724 remained after removing duplicates. After the initial title and abstract screening, 342 articles were identified for full text screening. Available full texts were screened for eligibility, resulting in the exclusion of 537 articles. Reasons for exclusion are provided (Figure 1). A total of 24 studies were included in our review.

An overview of all included studies (*n* = 24) is provided (Table 1). The earliest study included in our review was published in 2011, whilst remaining studies were published between 2012 and 2020. Various study designs were included, with randomised controlled trials (*n* = 10; 42%) being the most frequent. The most common diagnoses in which an adapted 6MWT was reported include respiratory disease (*n* = 8; 33%) and cardiovascular disease (*n* = 4; 17%). Studies were conducted predominantly in LMICs (*n* = 18; 75%), followed by four studies (17%) conducted in LIC (Benin, Malawi and Uganda), whilst two (8%) studies were conducted in a low-resource context of either an UMIC (Brazil) or HIC (Australia).

**TABLE 1:** Overview of included studies.

Source		Participants	6MWT reported purpose	ATS guideline variation						6MWD (m) baseline Mean (SD)	Rationale
Study design	Location setting	*n*; age (range) diagnosis		Length	Configuration	Venue	Instructions	Encouragement	Other		
Worringham et al. (2011) Pre-post	Australia‡ Metropolitan tertiary hospital & rural community health centre	Six adults (42–67) recent cardiac event or surgery	Evaluate pre-post impact of intervention on walking distance	NA	NA	Outdoor	NR	NR	Monitored by GPS to track distance walked	524 (NR)	Unable to attend conventional rehabilitation
Locks et al. (2012) RCT	Brazil‡ Physiotherapy school clinic	Eighty three adults (>60) healthy	Assess impact of intervention on cardiorespiratory fitness	20 m	Rectangle (9 m × 1 m)	NR	Continuously at a comfortable pace. Cover as much ground as possible in 6 min	NR	-	405 (62)	NR, the space that we had available to conduct the 6MWT§
Sen et al. (2012) Cross-Sectional	Kolkata India† Department of Paediatric Cardiology	Eighty one children (5–18) congenital cardiac disease	Evaluation of submaximal exercise capacity following surgical intervention	25 m	NR	In door	Walk briskly	NR	-	360 (82)	NR
Ralph et al. (2013) Case control	Papau Indonesia† Outpatient clinic	Two hundred adults pulmonary tuberculosis	Evaluate residual disability positive pulmonary TB results	NR	NR	Out door	ATS	NR	-	C: 497(63) I: 408(NR)	NR, did not have a long walking track available§
Rao et al. (2013) Cross-sectional	Pakistan† Medical Institute of Karachi	Two hundred and ninety six adults (15–65) healthy	Develop gender-specific predictive equations for healthy Pakistanis	18 m	Straight flat	In door	Cover as much distance as possible	Standard Protocol	-	470 (10)	NR
Zaky & Hassan (2013) RCT	Cairo Egypt† Hospital	Thirty children (8–12) Haemophilic knee arthritis	Determine effect intervention on functional ability	10 m	Rectangle	In door	ATS	NR	-	36 (11)	Available space
Mohamed et al. (2014) RCT	Egypt† Outpatient Chest Clinic	Thirty one adults (19–70) Chronic respiratory illnesses	Determine efficacy of intervention on 6MWD	NA	NA	NR	ATS	ATS	Electrical treadmill	32 (11)	Allows for constant monitoring
Nusdwinuringtyas et al. (2014) Cross-sectional	Indonesia† Department of Medical Rehabilitation Cipto Mangunkusumo Hospital	hundred and twenty three adults (18–50) healthy	Develop protocol-specific normative reference values	15 m	1st 6MWT protocol investigator developed	NR	NR	NR	-	547 (54)	NR, no 30 m distance§
					-	-	-	-	2nd 6MWT on Biodex gait trainer	545 (54)	
Sogbossi et al. (2014) Prospective cohort	Benin* Rehabilitation Centre	Two hundred and thirty adults Stroke	Validate against ABILOCO- Benin scale	50 m – 85 m	Square	NR	As quickly as possible	Informed of time at 2 min, 4 min, 5 min	-	NR	NR, contextual or / environmental restrictions across different testing centres§
Agrawal et al. (2015) Prospective cohort	India† Rural Based Teaching Hospital	Hundred and twenty nine adults >50 chronic respiratory disease	Determine the association of different factors and functional exercise capacity	46 m	Straight	In door	ATS	ATS	-	318 (89)	NR
Ben Saad et al. (2015) Case control	Tunisia† Farhat Hached Hospital	Two hundred and ninety adults (>40) obstructive sleep-apnoea-hypopnea-syndrome	To compare 6MWD between severe OSAHS patients (under CPAP treatment) with healthy controls	40 m	Straight	Out door	ATS	None	-	C: 624 (109) I: 531 (115)	NR
Guessogo et al. (2016) Prospective cohort	Cameroon† Jamot Hospital	Twenty eight adults pulmonary TB	Evaluate change in functional capacity during intervention	40 m	Straight long	NR	ATS	Time given every minute	-	C: 842(53) I: 572(121)	NR
Khan et al. (2016) RCT	New Delhi† Medical College and Associated Hospital	Sixty adults COPD	Evaluate impact of intervention on physical function	50 m	NR	In door	Standard	Standard	-	299 (18)	NR
Mahmoud et al. (2016) RCT	Egypt Cairo†	Forty adult men (50–60) Ischemic heart disease	Determine effect of intervention	44 m	NR	NR	Walk continuously covering as much ground as you can	NR	-	434 (3)	NR
Ranjita et al. (2016) RCT	India†	Eighty one adults (30–60) COPD	Determine effect of intervention on EC	35 m	Straight flat	In door	As much distance as possible	Standard phrases	-	302 (66)	NR
Agrawal et al. (2017) Pre-post	Mumbai India† Tertiary Health Care Centre	Eighty adults interstitial lung disease	Determine association of 6MWD (% predicted) with spirometry	24 m	Straight long	In door	ATS	NR	-	236 (NR)	NR
Daabis et al. (2017) RCT	Egypt† Hospital Department of Chest Disease	Forty five adults COPD	Evaluate if intervention is a useful addition in pulmonary rehabilitation	20 m	NR	NR	Standard protocol	NR	-	226 (107)	NR
Harikesavan et al. (2017) RCT	India† College of Physical Therapy Hospital	Eighteen adults (>50) total knee replacement	Impact of intervention on long-term functional performance	46 m	Rectangle	In door	As quickly as feels safe	NR	-	228 (71)	NR
Laing et al. (2017) RCT (cross-over)	Vietnam† Training Centre of Orthopaedic Technologies	Seventeen adults unilateral transtibial amputation	Impact of intervention on functional mobility	15 m	NR	In door	Walk at a normal comfortable speed	NR	-	320 (53)	NR, limited space available inside of the clinic §
Sims Sanyahumbi et al. (2017) cross-sectional	Malawi* Outpatient Clinic of Children’s Centre	Two hundred and twenty two children (4–18) HIV+	Compare exercise performance between HIV positive and healthy children	25 m	NR	Out door	Walk between cones for 6 min	NR	-	489 (521)	NR
Harikesavan et al. (2019) Prospective cohort	India† Manipal Hospital	Seventy eight adults >50 knee osteo-arthritis	Evaluate influence of intervention	46 m	Rectangular circuit	In door	As much distance as possible	NR	-	169 (70)	NR
Tripathi et al. (2019) RCT	India†	Sixty adults (18–45) healthy	Evaluate efficacy of intervention on physical performance	20 m	Straight	In door	At normal speed	NR	-	427 (42)	NR
Vancampfort et al. (2020a) cross-sectional	Kampala Uganda* Butabika Hospital	Fifty adults(18–65) major depressive disorder	Determine correlation with 2-min walk test	25 m	NR	Indoor	Walk back and forth around cones	ATS	-	395 (143)	NR
Vancampfort et al. (2020b) cross-sectional	Kampala Uganda* Butabika Hospital	Fifty adults (18–65) alcohol use disorder	Determine correlation with 2-min walk test	25 m	NR	Indoor	Walk back and forth around cones	ATS	-	480 (109.5)	NR

m, meters; NA, not applicable; NR, not reported;

* , low-income country;

† , low-to-middle income country;

‡ , conducted in a low-resource context of either high or upper-middle income country, Control; I, intervention; COPD, chronic obstructive pulmonary disease;

§ , additional information obtained through author correspondence;

6MWD, six-minute walk distance; ATS, American Thoracic Society; RCT, randomised control trial; SD, standard deviation; HIV, human immunodeficiency virus; OSAHS, obstructive-sleep-apnea-hypopnea-syndrome; EC, exercise capacity; CPAP, continuous-positive-airway-pressure; TB, tuberculosis.

### Six-minute walk test purpose

The majority of included studies (58%) reported using the 6MWT as a measure of submaximal exercise or functional capacity to determine the effect or effectiveness of an intervention in various pathologies. Other purposes of the 6MWT included validation of the 6MWT against other measures, (Nusdwinuringtyas et al. 2014; Sogbossi, Thonnard & Batcho 2014), correlation of 6MWT with 2-min walk test, (Vancampfort et al. 2020a, 2020b), developing normative values, (Nusdwinuringtyas et al. 2014; Rao et al. 2013) or as a descriptive variable in association with lung spirometry or QOL.

### Six-minute walk test adaptations

**FIGURE 2:**
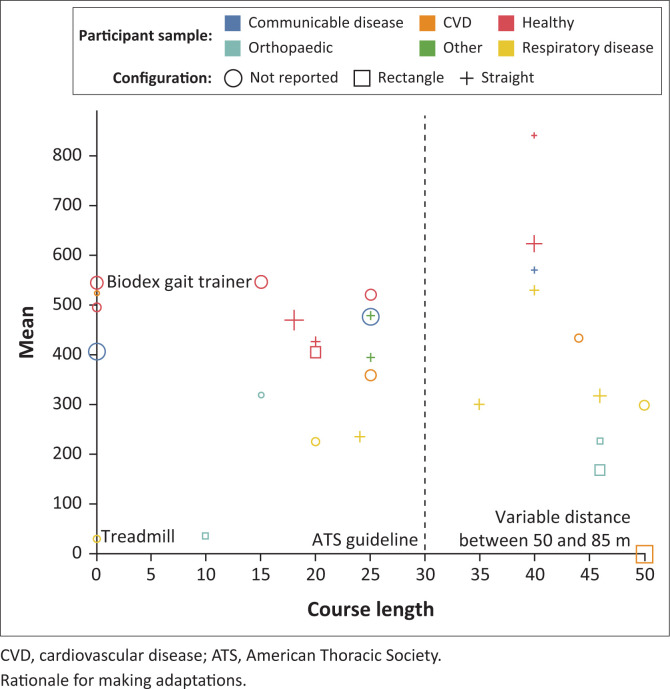
A graphical summary of the six-minute walk test variations for included studies (*n* = 24).

An overview of the adaptations made by the included studies is provided (Figure 2). The most common adaptation made to the ATS guidelines was that of course length (*n* = 21; 88%) ranging between 10 m and 85 m per lap. Of studies that adapted the course length, 12 (50%) reported a shorter pathway and 9 (38%) reported a pathway distance longer than 30 m. Twelve studies reported using a different configuration than straight, including using a rectangular (Harikesavan, Chakravarty & Maiya 2019; Harikesavan et al. 2017; Locks et al. 2012; Zaky & Hassan 2013) or square (Sogbossi et al. 2014) layout. One study reported using a 6MWT protocol developed by the study’s investigator (Nusdwinuringtyas et al. 2014). Four studies (17%) specifically reported conducting the 6MWT outdoors, 13 (54%) studies conducted the test indoors, as per ATS guidelines, whilst the remaining studies (*n* = 7; 29%) did not report detail on the test venue.

Instructions as per the ATS Guidelines were used in seven (29%) of the studies, whilst 13 (54%) studies reported instructions that were modified to include effort (e.g. walk briskly) or objective (e.g. cover as much distance as possible) (Rao et al. 2013).

The use of encouragement was generally not well reported, and standardised phrases were used (*n* = 7; 29%), whilst adapted (*n* = 2; 8%) encouragements included giving time at non-standardised time intervals (Sogbossi et al. 2014). Some studies refrained from using any encouragement during the test. Three studies included in this review reported ‘other’ adaptations which could all be linked to the use of technology. Nusdwinuringtyas et al. (2014) compared a conventional 6MWT (although 15 m course length) with one performed on a Biodex® gait trainer, reporting a non-significant mean difference of 2.73 m. Similarly, Mohamed et al. (2014) used a treadmill (controlled by the patient) to perform their test. Finally, Worringham et al. (2014) reported using global positioning system (GPS) monitoring to remotely track the distances walked by patients in a natural environment over the course of 6 min.

Eight of the included studies, five of which through author correspondence, provided a specific rationale for using an adapted 6MWT relative to a conventional ATS guideline version. Space constraint, as a result of environmental conditions, was the most common rationale for adapting the 6MWT (Laing et al. 2017; Locks et al. 2012; Nusdwinuringtyas et al. 2014; Ralph et al. 2013; Sogbossi et al. 2014; Zaky & Hassan 2013). Worringham, Rojek and Stewart (2011) reported conducting the test off-site for participants unable to attend standard rehabilitation sessions, whilst Mohammed et al. (2014) reported that using a treadmill (stationary test) allows for continuous monitoring of patient parameters.

## Discussion

To the best of our knowledge, this is the first study to explore adaptations to the ATS guidelines for conducting a 6MWT, in conjunction with the rationale for making these changes, specifically in LRS. Globally, LRS are seeing a significant, albeit slow, shift in disease burden from communicable disease towards non-communicable diseases (Murray et al. 2020). Because of the nature of the test, its applicability to assist in clinical decision-making, and its association with important outcomes like hospitalisation and mortality, there are compelling arguments for including the 6MWT as a key measure across the continuum of care. Standardisation in conducting the test is paramount from several perspectives including: the construct validity of the test as a measure of functional capacity, the within-patient homogeneity moving through various tiers of the healthcare system and ability to synthesise research outputs across settings for guideline development. Twenty-four studies were identified that predominantly reported variations in course length and configuration. In general, the methods used for conducting the 6MWT were poorly reported. Our review therefore assists in understanding the adaptations to the 6MWT ATS guidelines, drafted in 2002, made in LRS to inform the academic and clinical landscape and potentially consider the need for reconsidering these guidelines in the light of space constraints or other resource limitations (e.g. rurality).

In looking at the specific adaptations, space limitations specifically, were most commonly reported as a reason for shortening the course length. Unexpectedly, yet without clear reasons provided, almost half of studies included in this review reported using a *longer* (as opposed to *shorter)* course length, although, such a longer course length was often combined with a course reconfiguration (e.g. 80 m, square). Arguably, in studies where course length was increased and configuration altered, the course lay-out was adjusted pragmatically to adjust for the available space most likely to be interrupted. For instance, a short 10 m and straight course can be hypothesised to be more interruptive in that specific hallway compared to a rectangular 80 m ‘system of hallways’. The alternative to changing course length or configuration is to move the test from indoor to outdoor, as some studies in this review opted for (Ben Saad et al. 2015; Ralph et al. 2013; Sanyahumbi et al. 2017). Under reasonable weather conditions, no significant difference in 6MWD between indoor and outdoor 6MWT test settings has been reported, therefore opening up a possibly more feasible avenue to retain the standard ATS course configuration (Brooks et al. 2003).

The study samples in which the 6MWT was used were diverse, ranging from non-communicable disease to communicable disease, as well as mental health disorders, amongst others. The wide range of populations, beyond ‘cardiorespiratory’ indications, illustrates the wide use and value of the 6MWT for research and clinical practice. In most cases, the purpose for including the 6MWT was either as a measure of functional capacity to be associated with a second outcome measure (e.g. 6MWT vs. lung function, or 6MWT vs. the 2-min walk test), to allow for comparison between study samples, or as an outcome measure in studies of efficacy. The impact of diverting from the ATS standards can be considered different depending on the purpose for which the 6MWT was used, in particular in conjunction with reference values based on different configurations. In our review, two studies used (inadequate) reference equations for the interpretation of findings. Whilst, because pre-existing reference equations either over or underestimated the abilities of their patient population (Indonesian and Pakistani), two studies developed reference equations specific to their population anthropometrics. Additionally, one study in an Indian patient population found a significant association between the 6MWT and pulmonary hypertension when using the Indian reference equation. When the same analysis was completed using a different reference equation, correlation could not be determined. In an academic context, diverting from ATS guidelines might be less problematic, in particular in the context of pre-to-post testing when evaluating within patient or group changes over time. However, in clinical settings, this may lead to the lack of continuity as referred to earlier.

Reasons for adaptation were reported inconsistently, yet mostly pertained to space constraints. Interestingly, space constraints may not be endemic to LRS per se (albeit the implications in LRS may be more pronounced). For instance, Thaweewannakij et al. (2013) reported using a 6 m × 4 m rectangular walkway because of the absence of a large enough walkway in communities (Thailand, Upper-Middle Income Country), where the tests were conducted (Thaweewannakij et al. 2013). Furthermore, the notion of ‘LRS’ is more encompassing than a lack of space (Van Zyl et al. 2021). Barriers in service delivery, such as the logistics in accessing care, also need to be considered (Heller et al. 2019). In this light, the GPS-based 6MWT, to be used in a real-life environment, (Worringham et al. 2011) is a particularly interesting technological advancement, when moving towards telehealth and access to rural populations.

### Limitations

Our review has some limitations. Firstly, only studies that were published in English were considered. Secondly, the methods used for conducting the 6MWT, if reported, could often only be derived at full-text review stage. For many studies at full-text review, it was unclear as to the exact methods used. Given that some of the included studies in our review referred to the ATS guidelines as the 6MWT protocol, still reported adaptations thereof may indicate that adaptations were missed in those studies referring to the ATS guidelines with no further detail reported. Thirdly, additional adaptations could have been derived from studies conducted in high-resource settings not included in this review. Fourthly, it is also worth noting one specific adaptation to the 6MWT that was not considered in the scope of our review. The 2-min walk test or other adaptations to the duration of the test and its potential practical implications (e.g. time savings) were not studied. Finally, the impact of the adaptations on the primary outcome (6MWD) could not be derived from the data because of the underlying heterogeneity. Surprisingly, few studies reported validating their adapted 6MWT protocols against the gold standard.

## Conclusion

Space constraint is the most common reported reason for diverting from the ATS guidelines when using the 6MWT in LRS. Adaptations were made to the course length, or configuration to address these space limitations, often in conjunction. The ATS guidelines may need to be revisited, or context-specific norm values need to be developed, in order for the 6MWT to be more conducive to LRS (or other settings with space limitations).
